# Changes in the cortisol and oxytocin levels of first-time pregnant women during interaction with an infant: a randomized controlled trial

**DOI:** 10.1186/s12884-021-03609-8

**Published:** 2021-02-24

**Authors:** Nozomi Sonoda, Kaori Takahata, Wataru Tarumi, Kazuyuki Shinohara, Shigeko Horiuchi

**Affiliations:** 1 Japanese Red Cross Kyushu International College of Nursing, 1-1 Asty Munakata, Fukuoka, 811-4157 Japan; 2 Shonan Kamakura University of Medical Science, 1195-3 Yamasaki, Kamakura-shi, 247-0066 Japan; 3 Department of Neurobiology and Behavior, Graduate School of Biomedical Science, Nagasaki University, 1-12-4 Sakamotomachi, Nagasaki, 852-8523 Japan; 4 Graduate School of Nursing Science, St. Luke’s International University, 10-1 Akashi-cho, Chuo-ku, Tokyo, 104-0044 Japan

**Keywords:** Pregnancy, Primipara, Infant, Interaction, Cortisol, Oxytocin, Single nucleotide polymorphism, Randomized controlled trial (8/10)

## Abstract

**Background:**

During pregnancy, physiological, psychological, and social changes affect pregnant women’s childcare anxiety and childrearing behavior. However, there are scarce reports on hormonal evaluation related to such anxiety and behavior. Herein, we evaluated changes in salivary cortisol (primary outcome) and oxytocin (secondary outcome) levels of first-time pregnant women when interacting with an infant and discussed the relation of these changes to the women’s stress level.

**Methods:**

This was a two-arm randomized controlled trial. Participants were randomly assigned using a web-based randomization system. The experimental group involved interaction with an infant for 30 min. The control group involved watching a DVD movie of an infant for 30 min. Saliva samples were collected at preintervention and postintervention. Saliva samples were assayed, and all data were compared between and within the groups using independent t-test and paired t-test with a two-sided 5% significance level. This study was approved by the Research Ethics Committee of St. Luke’s International University.

**Results:**

A total of 102 women were randomly assigned to the experimental (n = 51) and control (n = 51) groups. Finally, 38 women in the experimental group and 42 women in the control group were analyzed. The salivary cortisol level significantly decreased after the interventions in both groups (t = 4.57, *p* = 0.00; t = 5.01, *p* = 0.00). However, there were no significant differences in the salivary cortisol (t = 0.349, *p* = 0.73) and oxytocin (t = − 1.945, *p* = 0.58) levels between the two groups.

**Conclusions:**

The salivary cortisol level of first-time pregnant women significantly decreased in the experimental and control groups postintervention, although no significant difference was found between the two groups. Such decrease indicates stress reduction and release among these women. The absence of a significant increase in salivary oxytocin level in both groups may be related to the limitations of an insufficient number of samples that could be analyzed owing to the small saliva volume in some samples and the lack of adequate tactile stimulation of the intervention protocol. These results and procedural limitations provide useful insights into approaching subsequent studies aiming at continuously optimizing detection procedures.

**Trial registration:**

UMIN000028471 (Clinical Trials Registry of University Hospital Information Network. July 31, 2017- Retrospectively registered.

**Supplementary Information:**

The online version contains supplementary material available at 10.1186/s12884-021-03609-8.

## Background

Over the past decades, the level of fertility of women has fallen globally [1]. In Japan, the total fertility rate has sharply declined to a rate of 1.4 live births per woman from 2015 to 2020 [2]. This serious decline in the total fertility rate has resulted in childcare anxiety in Japan. To address this concern, many Japanese municipalities provide various programs to women on how to rear their infants to reduce childcare anxiety based on the concept of “Healthy Parents and Children 21” [3]. However, effective infant interaction programs for Japanese pregnant women remain scarce.

During pregnancy, considerable physiological, psychological, and social changes occur which affect the childcare anxiety and childrearing behavior of pregnant women. In our previous study, we developed the “Mama’s Touch Program” which aimed to stimulate mother-baby bonding for first-time pregnant women through touching and holding infants [4]. This program consisted of two sections: section one involved interaction between the mother and the infant; (2) section two involved touching and holding of an infant by pregnant women [4]. The “Mama’s Touch Program” which involved touching and holding infants showed potential in decreasing the salivary cortisol level and altering the salivary oxytocin level [4].

Cortisol is widely known as a stress hormone. It is secreted from the adrenal cortex as a result of physical and mental stress stimuli, with increased levels observed 20 min after the stimuli [5–7]. Cortisol is detected in the blood and saliva with a 0.91 correlation between the sources [5], indicating that measurement of salivary cortisol level may provide a non-invasive method of assessing stress stimuli. Cortisol is used not only as a measure of stress but also as a measure of stress relief. Particularly in pregnant women, aromatherapy massage and yoga have been shown to reduce cortisol and relieve stress [8, 9].

On the other hand, oxytocin is widely known as an attachment and binding hormone. Oxytocin is synthesized in the neurons of the supraoptic nucleus in the hypothalamus and the paraventricular nucleus, secreted in pulses from the posterior pituitary into the blood, and found in the saliva and urine [10]. Oxytocin secretion is induced by nipple stimulation [11], touch and human-human relationship [12], and mother-infant interactions [13]. Furthermore, oxytocin secretion is associated with fetal attachment [14], child-rearing behavior [15], and preparation of women for motherhood [16]. Therefore, it is possible to clarify physiological changes in first-time pregnant women when they touch and hold an infant using cortisol and oxytocin levels.

We conducted a two-arm randomized controlled trial (RCT) to evaluate and compare changes in salivary cortisol and oxytocin levels of first-time pregnant women between experimental and control groups. The women in the experimental group touched and held an infant for 30 min (experimental intervention protocol), whereas those in the control group watched a DVD movie of an infant (control intervention protocol). The primary outcome was salivary cortisol level and the secondary outcome was salivary oxytocin level.

We hypothesize that at 30 min after touching and holding an infant, the salivary cortisol level will significantly decrease and the salivary oxytocin level will increase in the experimental group compared with the control group.

## Methods

### Participants and setting

The eligibility requirements were low-risk Japanese primiparas who 1) had singleton pregnancy, 2) were planning vaginal birth, 3) were over 20 years of age, and 4) could participate in the study between 38 weeks-0 days and 38 weeks-6 days of pregnancy. Excluded from the study were those who had any of the following conditions: 1) obstetric complication requiring treatment, 2) medical history of endocrine disorders or psychiatric disorders [5], 3) treatment for oral diseases including caries, 4) smoking habit [5], 5) nipple massage as self-care for birth induction [11], and 6) infant care involvement during pregnancy (Table 1). The present RCT was conducted in four facilities (i.e., hospital, clinic, birth center, perinatal medical center) in Tokyo, Japan between May 2017 and March 2018.

**Table 1:** Inclusion/exclusion criteria

Inclusion criteria	Exclusion criteria
1) Singleton pregnancy	1) Obstetric complication requiring treatment
2) Planning vaginal birth	2) Medical history of endocrine disorders or psychiatric disorders
3) > 20 years of age	3) Treatment for oral diseases including caries
4) Participate between 38 weeks-0 days and 38 weeks-6 days	4) Smoking habit
	5) Nipple massage as self-care for birth induction
	6) Infant care involvement during pregnancy

Sample size was determined based on a recent study by Sonoda et al. (2018) [4]. The present study had 80% power at the two tails of a 5% significance level to detect the mean difference (MD, 0.2 ng/mL) between two groups. The sample size was calculated as 72 women (36 women per group). The dropout rate was assumed to be 30%, thus the researchers planned to recruit 100 subjects before randomization. The reasons for dropping out were anticipated delivery and obstetric complications.

Participants were recruited from May 2017 to March 2018 in four facilities. Recruitment was stopped when 100 people needed before randomization were gathered and follow-up data were secured for the planned 36 women per group in March 2018. Participation was invited through the four cooperating facilities. After obtaining consent from these facilities, the study information and leaflet were posted at the outpatient wards before starting the recruitment.

Eligible pregnant women were selected by the researchers or institution staff. When eligible pregnant women after 34 weeks of gestation visited the hospital for a health check-up or a maternity class, verbal explanation of the objectives and methods of the study and an informed consent form were provided to the participants. To avoid discrepancies in the salivary cortisol and oxytocin level measurements, the participants were asked to abstain from 1) dental treatment two days before the study, 2) alcohol, spicy foods, and sexual intercourse the day before the study, 3) caffeine intake 12 h before the study, and 4) food intake one hour before the study. Upon agreement, two copies of the written informed consent form were signed, with one given to the participant and the other kept by the researchers.

The staff of the cooperating facilities introduced mothers and their infants to the research program, and 2- to 6-month-old infants and their mothers were recruited. When a mother and her infant visited the hospital for the one-month check-up or vaccination, verbal explanation of the study objectives and methods was provided using an informed consent form. Upon agreement to participate, two written informed consent forms were signed with one given to the mother and the other kept by the researchers.

### Ethics approval

This study was approved by the Research Ethics Committee of St. Luke’s International University, Tokyo, Japan (No. 17A-004) and was registered in the Clinical Trials Registry of University Hospital Information Network in Japan (UMIN000028471). This study was conducted in accordance with the “Ethical Guidelines on Medical Research for Human beings”. If a study-induced adverse event or a negative effect on the health of participants and infants occurred, appropriate treatment and other necessary measures were immediately taken. Any treatment was covered by the national health insurance system.

### Study design

This study was a two-arm RCT.

### Randomization and masking

At the time of agreement, the participants were randomly assigned to either the experimental group (intervention: touching and holding an infant) or the control group (intervention: watching a DVD movie of an infant) using a web-based randomization system, with permuted blocks of four.

The infants and mothers could not be masked because of the nature of the intervention. The biochemists who measured the salivary cortisol level (primary outcome) and salivary oxytocin level (secondary outcome) as physiological indices were blinded to the study.

### Procedures

The intervention was conducted between 9:00 and 15:00 in a quiet private room at the facility [5]. For the saliva collection method, all participants were instructed to 1) wash their mouth at the beginning of the study [17], 2) drink 100 mL of water 10 min before each saliva collection [18], and 3) pool their saliva at the bottom of the mouth for 3 min and then ease the pooled saliva into a cooled tube (passive drool method) [19, 20]. The volume of saliva needed for cortisol and oxytocin assay was 3.0 mL (i.e., 1.0 mL for cortisol assay and 2.0 mL for oxytocin assay). After washing the mouth and drinking 100 mL of water, each participant sat on a chair and watched a silent movie (Nature Therapy Seseragi, JAN 4961501647780, Della Inc., Japan) for 10 min to calm themselves and reduce any stress before saliva collection as the baseline (O1) value.

Saliva was collected at two points: before (O1) and after the intervention (O2). After saliva collection, the saliva samples were immediately stored in a freezer (Cryo Porter CS, Scinics Corp., Tokyo, Japan) at − 80 °C [18]. The saliva samples were transported under a frozen condition to the Physiology 2 Laboratory of the Graduate School of Medicine and Dentistry at Nagasaki University, Nagasaki, Japan and stored at − 80 °C before the cortisol and oxytocin assays.

Participants answered a questionnaire before (O1) and a questionnaire after (O2) the intervention following the saliva collection (Additional file 1-4). Additionally, buccal mucosa samples were obtained using a cotton swab for single nucleotide polymorphism (SNP: *rs*53576 and *rs*2254298) [21–23] assay of oxytocin using TaqMan assay (Applied Biosystems, Thermo Fisher, MA, USA) after completing the O2 questionnaire.

### Experimental group

The intervention protocol for first-time pregnant women in the experimental group involved the following activities:

1) *Observe the infant for 5 minutes*: make eye-to-eye contact with the infant, call the infant’s name, and touch the infant’s hand or leg

2) *Communicate with the infant for 10 min*: touch, sooth, talk, and make eye-to-eye contact following instructions from the mother.

3) *Hold the infant for 5 min*: hold the infant with eye-to-eye contact, and sooth and talk to the infant.

4) *Hold the infant for 5 min*: hold the infant after changing the position.

5) Observe the infant for 5 min: make eye-to-eye contact, call the infant’s name, and touch the infant’s hand or leg.

During the intervention, the following items were carefully noted: 1) crying and vomiting of the infant when under the care of the mother, and 2) non-acquisition of childcare skills of the participants such as failure to change the diapers or clothes.

To equalize the interventions, the mothers with their infant received training on how to interact with the pregnant women, and a third party checked the intervention every five to seven interventions**.**

### Control group

Using a similar situation as the experimental group, the participants in the control group watched a 30-min DVD movie of a 2-month-old girl in the following situations using a personal computer and headphones:

1) *The infant spending time quietly in her bed*: 15 min.

2) *The infant breastfeeding*: 4 min.

3) *The infant hugging her mother and finally falling asleep*: 5 min.

4) *The infant sleeping in her bed*: 6 min.

### Outcome measurements

#### Primary outcome: salivary cortisol level

Salivary cortisol level was analyzed using the Cortisol Salivary Immunoassay kit (Salimetrics, PA, USA) in the Physiology 2 Laboratory of the Graduate School of Medicine and Dentistry at Nagasaki University following the manufacturer’s instructions [24]. When the % coefficient of variation (%CV) of the inter-assay was > 10, the data was not used [24].

#### Secondary outcome: salivary oxytocin level

Salivary oxytocin level was analyzed by enzyme-linked immunosorbent assay (ELISA; ENZO Life Sciences, NY, USA) in the same laboratory following the manufacturer’s instructions [25]. Before the ELISA assay, the saliva sample was adjusted following the method of Carter et al. (2007) [26] and aprotinin was added (Sigma-Aldrich Corporation, MO, USA) [27]. When the %CV of the inter-assay was > 10, the data was not used [25].

#### Characteristics of participants

The questionnaire asked about the following items: height, weight, age, marital status, living with partner, have younger brother/sister, and experience of interactions with infants. The following data were obtained from the medical records: gestational date of birth, height, weight, age, and medical history. Basic information related to cortisol and oxytocin levels was also collected using a questionnaire.

SNP of oxytocin was assayed using buccal mucosa samples. Two oxytocin receptor gene polymorphisms (*rs*53576 and *rs*2254298) were assayed from the buccal mucosa samples by genotyping using TaqMan assay (Applied Biosystems).

### Statistical analysis

All data were analyzed using descriptive statistics and expressed as mean with standard deviation or frequency. The Shapiro-Wilk test was conducted for all O1 and O2 data, and histogram distributions were checked. Thereafter, the independent t-test was conducted for all data after the intervention (O2), and the amount of change was obtained as follows: ([O2 data] – [O1 data]). All outcomes in the experimental and control groups were compared within the group using the paired t-test. Statistical analysis was performed using SPSS Statistics version 25 (Static Base, Advanced Statistics, IBM Japan, Tokyo, Japan) with a two-sided 5% level of significance.

## Results

### Participant flow

**Fig. 1:**
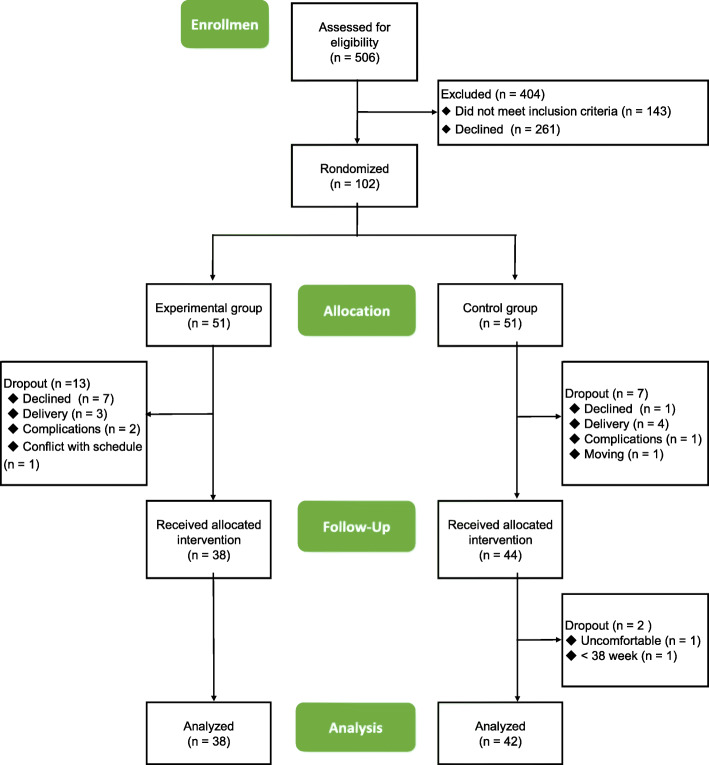
Participant flow diagram

The participant flow diagram is shown in Fig. 1**.** Of the 506 women who were eligible to participate, 102 women gave their informed consent and were randomly assigned using a web-based randomization system to one of the two groups: 51 women in the experimental group and 51 women in the control group.

Of the 51 women assigned to the experimental group, 13 women dropped out before the intervention. The reasons for dropping out were consent withdrawal (n = 7), birth before intervention (n = 3), obstetric complications (n = 2), and infection of the mother’s infant (n = 1). Finally, 38 women were analyzed in the experimental group; the dropout rate was 25.49%.

Of the 51 women assigned to the control group, seven participants dropped out before the intervention and two women dropped out by the end of the intervention. The reasons for dropping out before the intervention were consent withdrawal (n = 1), birth before intervention (n = 4), obstetric complications (n = 1), and transfer to another facility (n = 1). The reasons for dropping out by the end of the intervention were uncomfortable feeling during saliva collection (n = 1) and failure to satisfy the inclusion criteria during the intervention week (n = 1). Finally, 42 women were analyzed in the control group; the dropout rate was 17.65%.

### Baseline data

The characteristics of the participants are shown in Table 2. There was no significant difference between the two groups except for the state anxiety score. There were no participants who engaged in nipple stimulation to induce birth or had scars in their oral cavity.

**Table 2:** Baseline characteristics of patients

Characteristics			Experimental group *n* = 38		Control group *n* = 42	
Age (years)	*M*	[*SD*]	32.39	[4.59]	32.57	[4.32]
Body Mass Index before pregnancy	*M*	[*SD*]	20.95	[2.70]	20.78	[2.19]
Infertility treatment	*n*	(%)	8	(21.05%)	7	(16.67%)
Married	*n*	(%)	38	(100.00%)	41	(97.62%)
Having own younger brother/sister	*n*	(%)	23	(60.53%)	18	(42.86%)
Living with partner	*n*	(%)	38	(100.00%)	41	(97.62%)
Experience of interaction with infants	*n*	(%)	24	(63.16%)	18	(42.86%)
Delivery institution						
Hospital	*n*	(%)	17	(44.74%)	21	(50.00%)
Clinic	*n*	(%)	16	(42.11%)	13	(30.95%)
Birth center	*n*	(%)	5	(13.16%)	5	(11.90%)
Perinatal medical center	*n*	(%)	0	(0.00%)	3	(7.14%)
Salivary cortisol level	*M*	[*SD*]	4.60	[1.12]	4.77	[1.44]
State anxiety score	*M*	[*SD*]	36.97	[6.90]	40.33	[7.33]^*^
Trait anxiety score	*M*	[*SD*]	37.82	[7.21]	39.90	[6.73]
Salivary oxytocin level	*M*	[*SD*]	75.81	[47.37]	68.68	[46.15]
SNP rs2254298						
GG	*n*	(%)	19	(50.00%)	19	(45.24%)
GA	*n*	(%)	18	(47.37%)	16	(38.10%)
AA	*n*	(%)	1	(2.63%)	7	(16.67%)
SNP rs53576						
GG	*n*	(%)	5	(13.16%)	11	(26.19%)
GA	*n*	(%)	22	(57.89%)	14	(33.33%)
AA	*n*	(%)	11	(28.95%)	17	(40.48%)

### Primary outcome: salivary cortisol level

Salivary samples were collected at times O1 and O2 from the 38 participants in the experimental group and from the 42 participants in the control group. In one sample in the control group, salivary cortisol level at time O1 could not be analyzed owing to insufficient amount of saliva. In a duplicate assay, one sample in the experimental group at time O1 had a %CV of > 10, thus it was excluded from the statistical analysis. Therefore, the statistical analysis included 37 paired samples in the experimental group and 41 paired samples in the control group at times O1 and O2.

An independent t-test was conducted at time O2 between the experimental group (n = 37) and the control group (n = 41). The salivary cortisol level of the experimental group at time O2 was lower (4.01 ± 0.91 ng/mL) than that of the control group (4.11 ± 1.01 ng/mL), although there was no significant difference (t = − 0.459, *p* = 0.65). The amount of change in the cortisol level was not significantly different between the two groups **(**Table 3**).**

**Table 3:** Amount of changes in outcome measures

	Experimental group			Control group			t	*p*-value
	n	Mean	SD	n	Mean	SD		
Salivary cortisol level	37	−0.59	0.79	41	−0.66	0.84	0.349	0.73
Salivary oxytocin level	24	−15.40	21.03	24	−4.01	19.51	−1.945	0.58

**Fig. 2:**
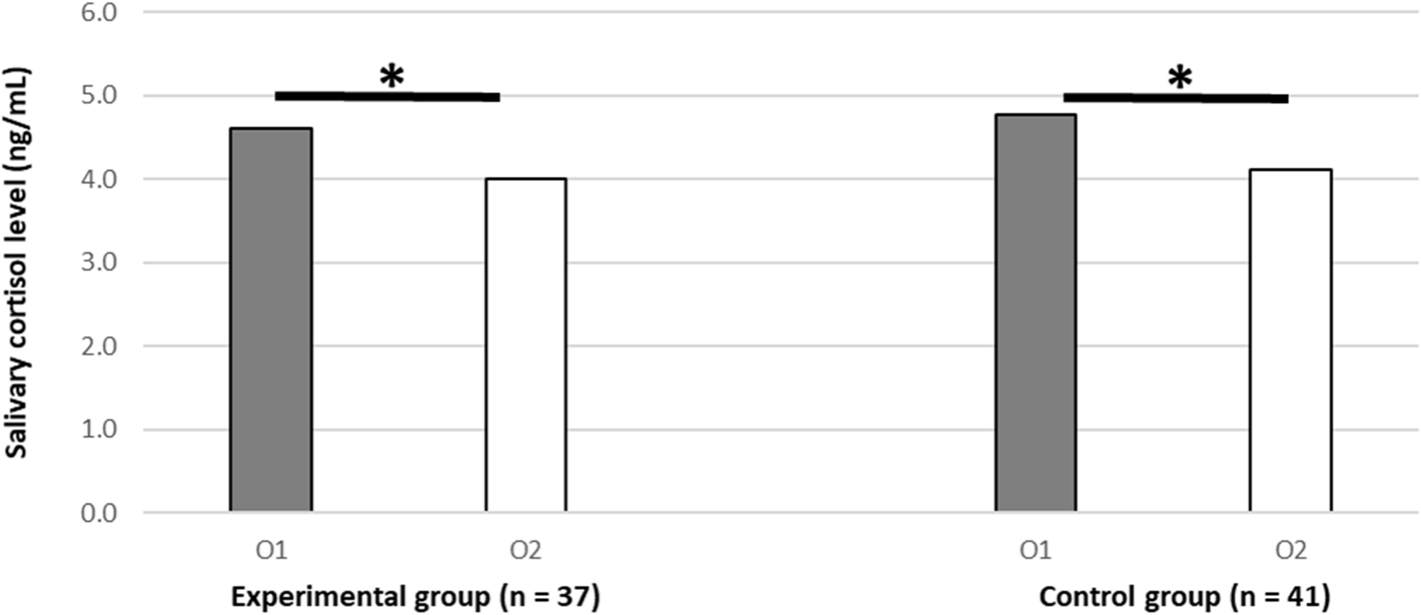
Changes in salivary cortisol level at O1 and O2 measurement point. O1, Before intervention. O2, After intervention

A paired t-test was conducted to compare the within-group salivary cortisol levels at times O1 and O2. In the experimental group (n = 37), the salivary cortisol level at time O1 was 4.60 ± 1.12 (ng/mL) and that at time O2 was 4.01 ± 0.91 (ng/mL). The salivary cortisol level at time O2 was significantly lower than that at time O1 (t = 4.570, *p* = 0.00). In the control group (n = 41), the salivary cortisol level at time O1 was 4.77 ± 1.44 (ng/mL) and that at time O2 was 4.11 ± 1.01 (ng/mL). The salivary cortisol level at O2 was significantly lower than that at O1 (t = 5.007, *p* = 0.00) **(**Fig. 2**)**.

### Secondary outcome: salivary oxytocin level

Salivary samples were collected at times O1 and O2 from the 38 participants in the experimental group and from the 42 participants in the control group. Some samples (14 samples in the experimental group and 18 samples in the control group) were excluded from the analysis owing to insufficient amount of saliva (i.e., saliva was < 2.0 mL or oxytocin was undetectable owing to high levels of impurities) and a %CV of > 10. Thus, statistical analysis was conducted on 24 samples at time O1 and in 24 samples at time O2 in both groups.

**Fig. 3:**
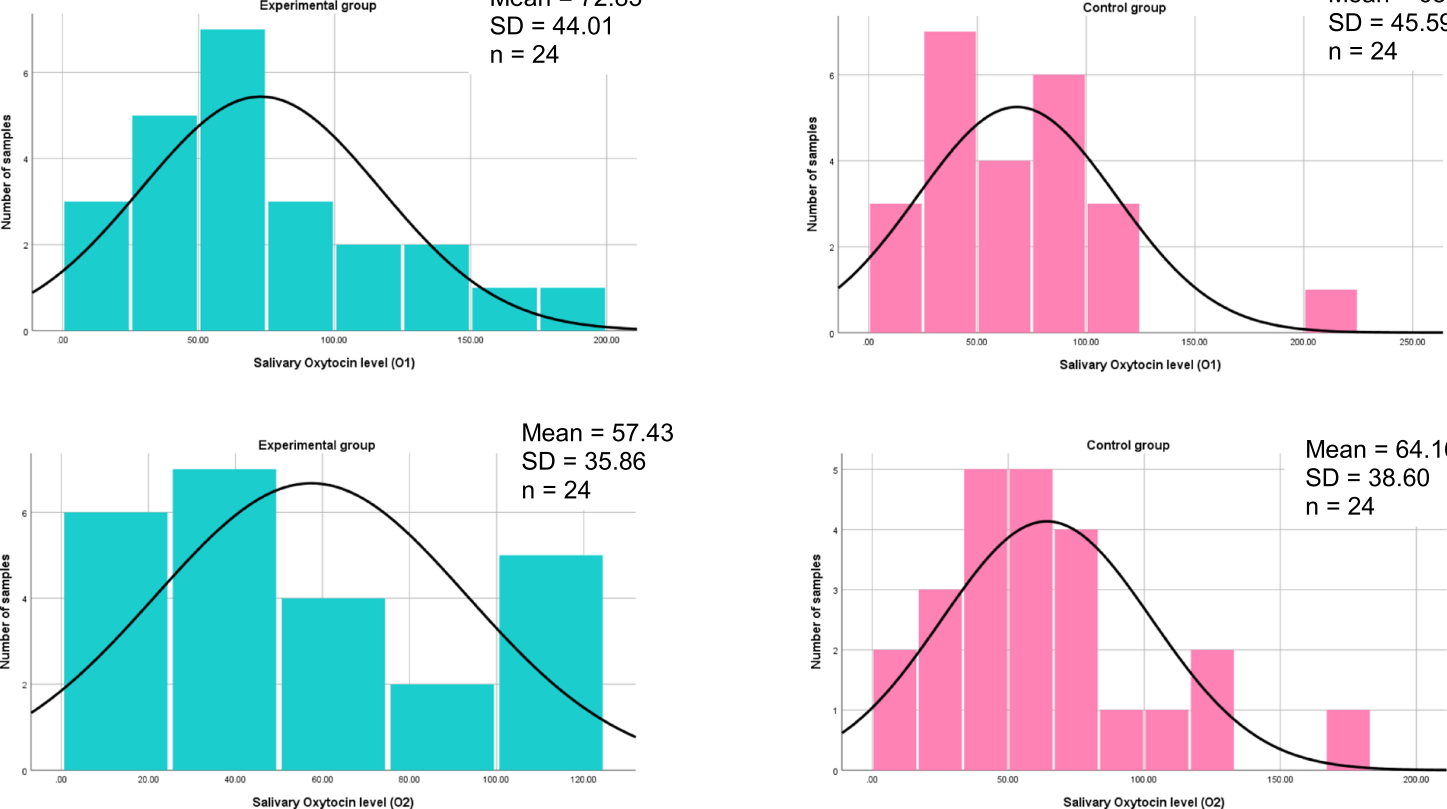
Histogram of salivary oxytocin level at O1 and O2 (experimental and control groups). O1, Before intervention. O2, After intervention

The distribution of salivary oxytocin level at O1 and O2 in both groups is shown in the histograms, and the range of oxytocin level was wide at 5.53–182.17 pg/mL at O1 and 7.03–121.70 pg/mL at O2 in the experimental group. Similarly, the range of oxytocin level at O1 was 15.09–224.09 pg/mL and that at O2 was 13.34–179.39 pg/mL in the control group (Fig. 3).

To compare the salivary oxytocin levels, an independent t-test was conducted at time O2 between the experimental group and the control group. The salivary oxytocin level in the experimental group at time O2 (n = 24) was 57.43 ± 35.86 (pg/mL) and that in the control group at time O2 (n = 24) was 64.16 ± 38.60 (pg/mL), with no significant difference (t = − 0.626, *p* = 0.53).

The amount of change in the experimental group was − 15.4 ± 21.0 (pg/mL) and that in the control group was − 4.01 ± 19.51 (pg/mL), with was no significant difference (t = − 1.945, *p* = 0.58) **(**Table 3**)**.

In the paired t-test, the salivary oxytocin level in the experimental group (n = 24) at time O1 was 72.83 ± 44.01 (pg/mL) and that at time O2 was 57.43 ± 35.86 (pg/mL). The salivary oxytocin level at time O2 was significantly lower than that at time O1 (t = 3.588, *p* = 0.00). In the paired t-test, the salivary oxytocin level in the control group (n = 24) at time O1 was 68.18 ± 45.59 (pg/mL) and that at time O2 was 64.16 ± 38.60 (pg/mL). There was no significant difference.

## Discussion

### Primary outcome: salivary cortisol level

This study showed a significant difference in the salivary cortisol levels after the interventions within the experimental group and the control group, but not between the two groups. As the number of subjects calculated by power analysis was met and the samples with a %CV of > 10 were excluded by duplicate assay, the results of this study are considered to be reliable and valid.

Cortisol is a biomarker whose level is increased at about 20 min after stress stimulation [5]. In this context, the salivary cortisol level represented biological changes in the first-time pregnant women in their late pregnancy as a result of interaction with an infant (experimental group) and watching a DVD movie of an infant (control group). The decrease in salivary cortisol levels after the interventions in both groups imply that these interventions were not stress stimuli to the first-time pregnant women.

Cortisol level has been reported to decrease with stress relief during pregnancy [8, 9, 28, 29]. Similarly in the present study, the salivary cortisol levels significantly decreased in both groups after the interventions. These results suggest that the interventions have the potential of inducing biological changes that are associated with stress relief.

However, there was no significant difference in the salivary cortisol level after the interventions between the experimental group and the control group. At this stage, there was no basis to clarify the stress reduction effect of the decrease in the cortisol levels between the experimental and control groups as the levels of decrease were not significantly different. Importantly, the exact mechanism of stress reduction in first-time pregnant women who interacted with an infant should be elucidated. Severi et al. (2005) examined changes in the salivary cortisol level of 12 pregnant women between 30 to 32 weeks of gestation after using the Fetouch system: 3D images using ultrasound and a system of interaction with the fetus using 3D images and haptic devices [30]. They showed that salivary cortisol level decreased in 11 of 12 pregnant women after the intervention [30]. In our previous pilot study, we compared the experimental group which interacted with infants and the control group which only watched a DVD movie of a landscape [4]. The salivary cortisol level in the experimental group was significantly decreased at 30 min after the intervention, whereas that in the control group was not [4]. In the present study, the results suggest that the decrease in salivary cortisol level in the control group was associated with stimulation from the infant images. Therefore, the specific effects of interacting with infants in terms of lowering the salivary cortisol level in both the experimental and control groups need further study and clarification. One could assert that the salivary cortisol level decreased with either interaction: being with an infant or viewing an infant on a DVD.

Notably, the present results suggest that the interaction of first-time pregnant women with an infant was not a stress stimulus, and that stress could be reduced by interacting with an infant or watching a DVD movie of an infant. Interacting with an infant presents many possibilities of improving the pregnancy period of women. Detailed clarification of the effects of interacting with an infant on stress reduction in mothers is warranted with due consideration of the appropriate intervention contents for the control group. This finding suggests that the effects of images of infants and mothers on salivary cortisol are the same as that of directly touching other people’s children. With the ongoing COVID-19 pandemic, stress reduction by the Web can produce the same results as real-life touch. This is especially important as effective tips from motherhood classes and face-to-face preparatory education are not currently possible during the COVID-19 pandemic.

### Secondary outcome: salivary oxytocin level

There was no significant difference in the salivary oxytocin levels between the two groups after the interventions. The salivary oxytocin levels in both groups did not increase after the interventions, which differed from our hypothesis. ELISA, which is a commonly used and rigorous method, was conducted in the present study to analyze salivary oxytocin level, and an inter-assay %CV of > 10 was not used. The data of salivary oxytocin level was assessed as reliable and valid.

Several underlying factors may be considered regarding the absence of an increase in salivary oxytocin level in the experimental group. First, there was an insufficient number of valid samples owing to the small volume of saliva for examining the oxytocin level. The volume of saliva needed to determine the salivary oxytocin level using ELISA is at least 2.0 mL. In the present study, 24 of the 38 samples in the experimental group and 24 of the 42 samples in the control group had a sufficient volume of saliva for analysis. Regarding the oxytocin detection method in the present study, the freeze-drying method was used to immediately centrifuge a thawed saliva sample. The centrifuged saliva was separated into a supernatant and a precipitate. When the proportion of the precipitate was large, the required amount of supernatant could not be obtained, making oxytocin measurement impossible. Oxytocin concentration varies greatly among individuals [11, 18]. Therefore, it is likely that a number of subjects in this study did not yield results. A larger volume of saliva and adequate number of valid samples would be necessary to ensure a definitive clarification of changes in the oxytocin level. Thus, there is a need continuously improve the analysis method at a given situation. Improvement in oxytocin detection procedures should also be a continuing goal in future assays. The recent oxytocin detection method of “Oasis HLB” has shown an effective extraction protocol. The protocol involves treatment with phosphoric acid phosphate, which has a protein-denaturing effect, followed by centrifugation to secure the required amount of supernatant. This improvement has succeeded in definitively detecting oxytocin [31]. The use of this protocol ensured the analysis of the oxytocin level in 14 out of 15 subjects [31]. These results show an example of a recent improvement in the oxytocin detection procedure that can be considered for future studies.

Second, the experimental intervention protocol may also have a limitation in that there may not be sufficient stimulation from touching and holding of an infant as well as the appropriate stimulus for oxytocin secretion. Thus, although the interventions may have lowered the salivary cortisol level, they did not sufficiently increase the salivary oxytocin level. This may be related to C-tactile afferents (CTs) which are present in the tactile receptors of the skin that triggers oxytocin release upon their activation [32]. CTs respond to an innocuous skin temperature stimulus moving with low force and velocity (1–10 cm/s).

Part of the important contributions of this research are showing the limitations of the current oxytocin detection method and protocol used and providing recommendations for improvements. In future studies, a larger amount of saliva, an adequate number of samples, and an experimental intervention protocol that achieves tactile stimulation over wider areas to activate CTs would reduce procedural limitations and ensure optimal outcome measurements.

### Limitations of this study

The first limitation is related to the protocol of watching a DVD movie of an infant in the control group. Although the DVD movie did not stimulate the tactile or olfactory sense, it may have had the same effect of cortisol level reduction in the experimental group brought about by the calm image of the infant in the movie. The second limitation is related to insufficient amount of collected saliva in some sample and the lack of appropriate tactile stimulation to activate CTs.

## Conclusions

The present two-arm RCT clarified some physiological changes between first-time pregnant women who had a 30-min interaction with an infant (experimental group) and first-time pregnant women who watched a 30-min DVD movie of an infant (control group). The women showed a significant decrease in salivary cortisol levels after the interventions within both groups, but not between the two groups. The absence of a significant difference in the salivary oxytocin level in both groups may be related to the limitations of an insufficient number of valid samples that could be analyzed owing to the small volume of saliva or supernatant in some samples and the lack of adequate tactile stimulation of the current protocol. These results and procedural limitations provide useful insights into identifying the strengths, weaknesses, and factors that produce contrasting results to previous studies and recommendations for improvements of oxytocin detection procedures in subsequent studies for the benefit of pregnant women.

## Supplementary Information

**Additional file 1.** Questionnaire before the intervention (English language version).

**Additional file 2.** Questionnaire after the intervention (English language version).

**Additional file 3.** Questionnaire before the intervention (Japanese version).

**Additional file 4.** Questionnaire after the intervention (Japanese version).

**Additional file 5.** CONSORT 2010 checklist

## Data Availability

The datasets used and analyzed in the present study are available from the corresponding author upon reasonable request.

## Abbreviations

RCT: Randomized Controlled Trial
SNP: Single Nucleotide Polymorphism
