# Supplementary material for: Changes in the cortisol and oxytocin levels of first-time pregnant women during interaction with an infant: a randomized controlled trial
Source: BMC Pregnancy Childbirth. 2021 Feb 24;21:162. doi: 10.1186/s12884-021-03609-8 (PMC7903931; doi:10.1186/s12884-021-03609-8)
Supplement: Supplementary file 1 — Additional file 1. Questionnaire before the intervention (English language version). [file 12884_2021_3609_MOESM1_ESM.docx]

Study date: month/day/year

　　　　　　　　　　　　　　　　　　　　　　　　　　　　　　　　　ID

**Thank you for taking part in this study.**

**The questionnaire has 8 pages and takes approximately 20 minutes to complete.**

**Please read the instructions before answering the questions.**

**This questionnaire is only related to the content of the program and content related to the hormone being measured.**

**Ⅰ．**Please answer the following questions about yourself. For multiple-choice questions, please circle the number that best applies to you.

| 1. Expected date of delivery | (Month) (Day) |
| --- | --- |
| 2. Current week of pregnancy | (Week(s)) (Day(s)) |
| 3. Your baby’s gender | (1) Boy  (2) Girl  (3) I don’t know, yet.  (4) I don't want to know until he/she will born. |
| 4. Height | cm |
| 5. Weight at your most recent prenatal checkup | kg |
| 6. Age | years |
| 7. Marital status | (1) Married  (2) Unmarried |
| 8. Status of cohabitation with your partner | (1) Cohabitating  (2) Living separately |
| 9. Is this your first pregnancy? | (1) Yes  (2) No |
| 10. Do you have any younger brothers or sisters? | (1) Yes  (2) No |
| 11. Have you ever cared for a baby before? | (1) Yes ≫≫≫≫≫≫≫≫≫≫ Go to 12.  (2) No ≫≫≫≫≫≫≫≫≫≫ Go to 13. |
| 12-1. Who did you care for?  *Multiple responses are acceptable | (1) Younger brother or sister  (2) Relative’s child  (3) Neighbor’s child  (4) Friend’s child  (5) Other  ( ) |
| 12-2. What kind of care did you provide?  *Multiple responses are acceptable | (1) Held in my arms/carried on my back  (2) Changed diaper(s)  (3) Fed milk  (4) Bathed  (5) Cradled/played with  (6) Other  ( ) |
| 12-3. How often did you provide care? | (1) Only once  (2) Once or twice a month  (3) Once or twice a week  (4) Three or four times a week  (5) Every day |
| 13. What kind of image do you have of “babies”? |  |
| 14-1. Are you worried about living with a baby? | (1) Yes  (2) No |
| 14-2. If you answered (1) Yes, please describe what kind of worries you have. |  |
| 15．Do you have anxiety other than living with a baby? | (1) Anxiety about delivery  (2) Anxiety about family relationship  (3) Anxiety about economic conditions  (4) Other  (　　　　) |
| 16-1. Are you doing anything to prepare for childbirth? | (1) Yes  (2) No |
| 16-2. If you answered (1) Yes, please describe what preparations you are making. |  |
| 17．How do you feel at the moment? | (1) Excited  (2) As usual  (3) Restless |

**Ⅱ．**

State anxiety score was measured by **STAI-form JYZ**. Hidano, et al. (2000), developed the STAI-form JYZ and achieved a confirmed Cronbach α of .859-.923. State anxiety score consists of 20 items with a 4-point Likert scale. State anxiety score’s range was 20-80, and 20-45 was judged as low anxiety and >55 was judged as high anxiety.

**Ⅲ．**

Trait anxiety was assessed using **STAI-form JYZ**, with a confirmed with Cronbach α .859-.923. Trait anxiety score consisting of 20 items has a 4-point Likert scale. Trait anxiety score’s range was 20-80, and 20-45 was judged as low anxiety and over 55 was judged as high anxiety.

Hidano T, Hukuhara M, Iwawaki S, Soga S, Spielberger CD. Manual of State-Trait Anxiety Inventory-From JYZ [Translated from Japanese.]. Jitsumukyouiku shuppan. 2000.

**Ⅳ．**

Depression was assessed using the **Japanese version of PHQ-9** (Muramatsu, et al, 2009), which consisted of nine items with a 4-point Likert scale. Muramatsu, et al. (2007) truncated the PHQ: Patient Health Questionnaire for Japanese, which then had a sensitivity 0.84, specificity 0.95, positive predictive value 0.87, negative predictive value 0.94 and kappa coefficient 0.79. A total score over 10 was classified as moderate severe to severe depression.

Muramatsu K, Muyaoka H, Muramatsu Y, Yoshida M, Ostubo T, Gejyo F. The patient health questionnaire, Japanese version: Validity according to the mini-international neuropsychiatric interview-plus. Psychological Reports. 2007: 952-960.

**Ⅴ．**

Experiences of parental bonding were assessed using the **Japanese version of Parental Bonding Instrument** (PBI). PBI was developed by Parker (1979) and shortened in the Japanese version by Ogawa (1991). It has two scales: care factor and over-protection factor. Each has confirmed validity for content validity, construct validity and concurrent validity and reliability (Cronbach’s alpha was 0.83-0.92). Care factor (CA) consists of 12 items and over-protection factor (OP) consists of 13 items with a 4-point Likert scale. High score of care factor means the person received warm-caring from their caregivers, and high score of over protection factor means the person received over protected care from their care givers.

Masami O. Verification of Reliability and Validity of PBI (Parental Bonding Instrument); Japanese version [Translated from Japanese.]. Japanese Journal of Psychiatric Treatment. 1991; 6: 1193-1201.

**Ⅵ．**

***Taiji Kanjyo Hyoutei Shakudo*** could measure feelings about the image or imagination of the infant. *Taiji Kanjyo Hyoutei Shakudo* was developed by Hanazawa (1992). It has two scales, namely, approach feeling and avoidance feeling, each consisting of 14 items with a 4-point Likert scale. Approach feeling indicates the positive feelings for the infant (e.g. joyful, cheerful and beautiful), and the higher score means more positive feelings for infant. Avoidance feeling indicates the negative feeling for the infant, and the higher score means more negative feelings for the infant (e.g. noisy, bothersome and frightening). The highest score for each scale is 42. The approach feeling and avoidance feeling were confirmed in terms of validity (checked correlation of question of infants, Approach feeling: *r* = .76, Avoidance feeling: *r* = .68) and reliability (checked by re-test method, Approach feeling: *r* = .85, Avoidance feeling: *r* = .85).

Hanazawa S. Maternal Psychology [Translated from Japanese.]. Igakushoin. 1992.

●This is the end of the questionnaire●
