# Supplementary material for: Changes in the cortisol and oxytocin levels of first-time pregnant women during interaction with an infant: a randomized controlled trial
Source: BMC Pregnancy Childbirth. 2021 Feb 24;21:162. doi: 10.1186/s12884-021-03609-8 (PMC7903931; doi:10.1186/s12884-021-03609-8)
Supplement: Supplementary file 2 — Additional file 2. Questionnaire after the intervention (English language version). [file 12884_2021_3609_MOESM2_ESM.docx]

Study date: month/day/year

ID

**Thank you for taking part in this study.**

**The questionnaire has 4 pages and takes approximately 10 minutes to complete.**

**Please read the instructions before answering the questions.**

Ⅰ．Please answer the following questions. For multiple-choice questions, please circle the number that best applies to you.

| 1. Has your image of babies changed after taking part in the study compared with before?  If you answered (1) Changed, please describe how your image has changed. | (1) Changed  (2) Not changed |
| --- | --- |
| 2. Have your worries about living with a baby changed after taking part in the study compared with before?  If you answered (1) Changed, please describe how your worries have changed. | (1) Changed  (2) Not changed |
| 3．Have your anxiety other than about living with a baby changed before and after participating in the study? | (1) Increase anxiety about delivery  (2) Decrease anxiety about delivery  (3) Increase anxiety about family relationship  (4) Decrease anxiety about family relationship  (5) Increase anxiety about economic conditions  (6) Decrease anxiety about economic conditions  (7) Other: Increase (　　　　)  (8) Other: Decrease (　　　　) |
| 4. Do you want to join the program again? | (1) Yes  (2) No |
| 5．Have you felt burdened or anxious in this program?  If you answered (1) Yes, please describe how your burden or anxious have felt. | (1) Yes  (2) No |
| 6．Please describe your think about this program. |  |

**Ⅱ．**

State anxiety score was measured by **STAI-form JYZ**. Hidano, et al. (2000), developed the STAI-form JYZ and achieved a confirmed Cronbach α of .859-.923. State anxiety score consists of 20 items with a 4-point Likert scale. State anxiety score’s range was 20-80, and 20-45 was judged as low anxiety and >55 was judged as high anxiety.

Hidano T, Hukuhara M, Iwawaki S, Soga S, Spielberger CD. Manual of State-Trait Anxiety Inventory-From JYZ [Translated from Japanese.]. Jitsumukyouiku shuppan. 2000.

**Ⅲ．**

***Taiji Kanjyo Hyoutei Shakudo*** could measure feelings about the image or imagination of the infant. *Taiji Kanjyo Hyoutei Shakudo* was developed by Hanazawa (1992). It has two scales, namely, approach feeling and avoidance feeling, each consisting of 14 items with a 4-point Likert scale. Approach feeling indicates the positive feelings for the infant (e.g. joyful, cheerful and beautiful), and the higher score means more positive feelings for infant. Avoidance feeling indicates the negative feeling for the infant, and the higher score means more negative feelings for the infant (e.g. noisy, bothersome and frightening). The highest score for each scale is 42. The approach feeling and avoidance feeling were confirmed in terms of validity (checked correlation of question of infants, Approach feeling: *r* = .76, Avoidance feeling: *r* = .68) and reliability (checked by re-test method, Approach feeling: *r* = .85, Avoidance feeling: *r* = .85).

Hanazawa S. Maternal Psychology [Translated from Japanese.]. Igakushoin. 1992.

●This is the end of the questionnaire●
