# Supplementary material for: Changes in the cortisol and oxytocin levels of first-time pregnant women during interaction with an infant: a randomized controlled trial
Source: BMC Pregnancy Childbirth. 2021 Feb 24;21:162. doi: 10.1186/s12884-021-03609-8 (PMC7903931; doi:10.1186/s12884-021-03609-8)
Supplement: Supplementary file 3 — Additional file 3. Questionnaire before the intervention (Japanese version). [file 12884_2021_3609_MOESM3_ESM.docx]

研究日year/month/day

ID

**研究にご参加いただき、ありがとうございます。**

**質問紙は8ページあり、所要時間は約20分です。**

**説明を読んで頂き、ご回答をよろしくお願いいたします。**

**なお、この質問紙は、プログラムの内容に関すること、**

**測定するホルモンに関連する内容のみになっています。**

**Ⅰ．**あなたご自身のことについて、以下の質問にご記入をお願いいたします。選択肢があるものに関しては、あてはまる数字を◯で囲んでください。

| 1. 分娩予定日 | 月　　日 |
| --- | --- |
| 2. 今日の妊娠週数 | 週　　日 |
| 3．お腹の赤ちゃんの性別 | ①　男の子  ②　女の子  ③　知りたいが、まだ知らない  ④　生まれてからの楽しみ |
| 4. 身長 | cm |
| 5. 最近の妊婦健診での体重 | Kg |
| 6. 年齢 | 歳 |
| 7. 婚姻状態 | ①　既婚  ②　未婚 |
| 8. パートナーとの同居 | ①　同居  ②　別居 |
| 9. 今回の妊娠は初めての妊娠ですか？ | ①　はい  ②　いいえ |
| 10. あなたに、弟もしくは妹はいますか？ | ①　いる  ②　いない |
| 11. あなたは今まで、赤ちゃんのお世話をしたことが  　　ありますか？ | ①　ある　≫≫≫≫≫≫≫≫　12.へ  ②　ない　≫≫≫≫≫≫≫≫　13.へ |
| 12-1. 誰のお世話をしましたか？  　　　＊複数回答可 | ①　弟や妹  ②　親戚の子ども  ③　近所の子ども  ④　友人の子ども  ⑤　その他（　　　　　　　） |
| 12-2. どのようなお世話をしましたか？  　　　＊複数回答可 | ①　抱っこ・おんぶ  ②　おむつを替える  ③　ミルクを飲ませる  ④　お風呂に入れる  ⑤　あやす・遊ぶ  ⑥　その他（　　　　　　　） |
| 12-3. お世話の頻度はどれくらいでしたか？ | ①　1回だけ  ②　月に1〜2回  ③　週に1〜2回  ④　週に3〜4回  ⑤　毎日 |
| 13. 「赤ちゃん」はどのようなイメージですか？ |  |
| 14-1. 赤ちゃんとの生活や育児に不安はありますか？ | ①　ある  ②　ない |
| 14-2. ①ある と答えた方はどのような不安がありますか？ |  |
| 15．赤ちゃんとの生活や育児の他にも不安はありますか？ | ①　お産に関すること  ②　家族との関係  ③　経済的なこと  ④　その他（　　　　　　　　　） |
| 16-1.お産に向けて、何かしていることはありますか？ | ①　ある  ②　ない |
| 16-2.①ある と答えた方は何をしていますか？ |  |
| 17．いま、現在の気持ちはどのような気持ちですか？ | ①　ワクワクしている  ②　いつも通り  ③　落ち着かない |

**Ⅱ．**

**新版STAI-From JYZ**を用い、状態不安得点の測定を行った。新版STAI-From JYZは、肥田野他(2000) によって開発された尺度で、Cronbach α係数は .859から.923である。状態不安尺度は、対象者が“今まさにどのように感じているか”を評価する不安存在尺度と不安不在尺度の20の叙述文から成り立っており、各項目は1点から4点までの重みづけがされている。状態不安尺度の得点は20点から80点までの間に分布し、段階1から段階5に分類される。20点以上45点未満は低不安、55点以上は高不安と判定される。

**Ⅲ．**

**新版STAI-From JYZ**を用いて、特性不安得点の測定を行った。新版STAI-From JYZは、肥田野他(2000)によって開発された尺度で、Cronbach α係数は .859から.923である。特性不安尺度は、“ふだん一般にどのように感じているか”を査定する不安存在尺度と不安不在尺度の20の叙述文から成り立っている。各項目は、1点から4点までの重みづけがされている。特性不安尺度の得点は20点から80点までの間に分布し、20点以上45点未満は低不安、55点以上は高不安と判定される。

肥田野直, 福原眞知子, 岩脇三良, 曽我祥子, Spielberger (2000). 新版STAIマニュアル, 東京: 実務教育出版.

**Ⅳ．**

**PHQ-9日本語版**を用い、抑うつに関して情報を得た。PHQ-9は、PHQ: Patient Health QuestionnaireはPrimary Care Evaluation of Medical Disorderをもとに作成された精神疾患の評価ツールで、うつ病性障害に関わる9つの質問項目を抽出して作成された質問紙である。PHQ-9日本語版は、村松他 (2009) によって翻訳され、感度0.84、特異度0.95、陽性的中率0.87、陰性的中率0.94、kappa係数0.79となっている。PHQ-9は過去2週間の症状について「全くない」「数日」「半分以上」「ほとんど毎日」の4段階で回答し、合計点により症状評価ができる。合計点が1から4点は軽微、5から9点は軽度、10から14点は中等度、15から10点は中等度から重度、20点以上は重度と分類される。

Muramatsu K, Muyaoka H, Muramatsu Y, Yoshida M, Ostubo T, Gejyo F. The patient health questionnaire, Japanese version: Validity according to the mini-international neuropsychiatric interview-plus. Psychological Reports. 2007: 952-960.

新潟青陵大学大学院臨床心理学研究, 第7号, p35-39, 2014.

**Ⅴ．**

**Parental Bonding Instrument (PBI)日本版**を用い、被養育体験に関して情報を得た。PBIはParker(1979)によって作成され、小川(1991)によって日本語版が作成され、信頼性・妥当性の確認が行われた。PBI日本版も12項目の養護因子(care factor)と13項目の過保護因子(over-protection factor)からなり「まったく違う（0点）」から「非常にそうだ（3点）」の4件法で評価される。

小川雅美(1991). PBI(Parental Bonding Instrument)日本版の信頼性, 妥当性に関する研究. 6(10), 1193-1201.

**Ⅵ．**

**対児感情評定尺度**を用い、乳児に対するイメージを得点化した。対児感情尺度は花沢 (1992) によって作成された尺度で、接近得点と回避得点、各14項目からなる赤ちゃんに対するイメージを4件法にてスコア化したものである。両項目とも最高点は42点である。信頼性に関しては再検査法にて検証され (接近得点*r* = 0.85、回避得点*r* = 0.85) 、妥当性に関しては乳児に関する質問との相関にて検証されている (接近得点*r* = 0.76、回避得点*r* = 0.68) 。

花沢成一(1992). 母性心理学, 東京: 医学書院.

●質問は以上になります●
