# Supplementary material for: Changes in the cortisol and oxytocin levels of first-time pregnant women during interaction with an infant: a randomized controlled trial
Source: BMC Pregnancy Childbirth. 2021 Feb 24;21:162. doi: 10.1186/s12884-021-03609-8 (PMC7903931; doi:10.1186/s12884-021-03609-8)
Supplement: Supplementary file 4 — Additional file 4. Questionnaire after the intervention (Japanese version). [file 12884_2021_3609_MOESM4_ESM.docx]

研究日year/month/day

ID

**研究にご参加いただき、ありがとうございました。**

**質問紙は4ページあり、所要時間は約10分です。**

**説明を読んで頂き、ご回答をよろしくお願いいたします。**

Ⅰ．以下の質問にご記入をお願いいたします。選択肢があるものに関しては、あてはまる数字を◯で囲んでください。

| 1. 研究参加前と参加後を比較して、赤ちゃんのイメージは変化しましたか？  イメージが ①変化した方は、どのようなイメージになったかご記入ください。 | ①　変化した  ②　変化していない |
| --- | --- |
| 2. 研究参加前と参加後を比較して、赤ちゃんとの生活や育児への不安は変化しましたか？  不安が ①変化した方は、どのように変化しましたか。内容をご記入ください。 | ①　変化した  ②　変化していない |
| 3．研究参加前と参加後を比較して、赤ちゃんとの生活や育児以外の不安は変化しましたか？  　 当てはまるものを選んでください。 | ①　お産に関する不安が増えた  ②　お産に関する不安が減った  ③　家族との関係に関する不安が増えた  ④　家族との関係に関する不安が減った  ⑤　経済的な不安が増えた  ⑥　経済的な不安が減った  ⑦　その他（　　　　　　　　　）が増えた  ⑧　その他（　　　　　　　　　）が減った |
| 4. このようなプログラムがあれば、参加したいと思いますか？ | 1. はい 2. いいえ |
| 5．このプログラムに参加して、負担を感じたことや不安を感じたことはありますか？  　 ①はい　の方は、その内容をご記入下さい。 | 1. はい   ② いいえ |
| 6．プログラムに関する感想などお書き下さい。 |  |

**Ⅱ．**

**新版STAI-From JYZ**を用い、状態不安得点の測定を行った。新版STAI-From JYZは、肥田野他(2000) によって開発された尺度で、Cronbach α係数は .859から.923である。状態不安尺度は、対象者が“今まさにどのように感じているか”を評価する不安存在尺度と不安不在尺度の20の叙述文から成り立っており、各項目は1点から4点までの重みづけがされている。状態不安尺度の得点は20点から80点までの間に分布し、段階1から段階5に分類される。20点以上45点未満は低不安、55点以上は高不安と判定される。

肥田野直, 福原眞知子, 岩脇三良, 曽我祥子, Spielberger (2000). 新版STAIマニュアル, 東京: 実務教育出版.

**Ⅲ．**

**対児感情評定尺度**を用い、乳児に対するイメージを得点化した。対児感情尺度は花沢 (1992) によって作成された尺度で、接近得点と回避得点、各14項目からなる赤ちゃんに対するイメージを4件法にてスコア化したものである。両項目とも最高点は42点である。信頼性に関しては再検査法にて検証され (接近得点*r* = 0.85、回避得点*r* = 0.85) 、妥当性に関しては乳児に関する質問との相関にて検証されている (接近得点*r* = 0.76、回避得点*r* = 0.68) 。

花沢成一(1992). 母性心理学, 東京: 医学書院.

●質問は以上になります●
